# Seizure-Related Gene 6 (Sez-6) in Amacrine Cells of the Rodent Retina and the Consequence of Gene Deletion

**DOI:** 10.1371/journal.pone.0006546

**Published:** 2009-08-07

**Authors:** Jenny M. Gunnersen, Annabel Kuek, Joanna A. Phipps, Vicki E. Hammond, Theresa Puthussery, Erica L. Fletcher, Seong-Seng Tan

**Affiliations:** 1 Brain Development, Howard Florey Institute, University of Melbourne, Parkville, Victoria, Australia; 2 Department of Anatomy & Cell Biology, University of Melbourne, Parkville, Victoria, Australia; Institut de la Vision, France

## Abstract

**Background:**

Seizure-related gene 6 (Sez-6) is expressed in neurons of the mouse brain, retina and spinal cord. In the cortex, Sez-6 plays a role in specifying dendritic branching patterns and excitatory synapse numbers during development.

**Methodology/Principal Findings:**

The distribution pattern of Sez-6 in the retina was studied using a polyclonal antibody that detects the multiple isoforms of Sez-6. Prominent immunostaining was detected in GABAergic, but not in AII glycinergic, amacrine cell subpopulations of the rat and mouse retina. Amacrine cell somata displayed a distinct staining pattern with the Sez-6 antibody: a discrete, often roughly triangular-shaped bright spot positioned between the nucleus and the apical dendrite superimposed over weaker general cytoplasmic staining. Displaced amacrines in the ganglion cell layer were also positive for Sez-6 and weaker staining was occasionally observed in neurons with the morphology of alpha ganglion cells. Two distinct Sez-6 positive strata were present in the inner plexiform layer in addition to generalized punctate staining. Certain inner nuclear layer cells, including bipolar cells, stained more weakly and diffusely than amacrine cells, although some bipolar cells exhibited a perinuclear “bright spot” similar to amacrine cells. In order to assess the role of Sez-6 in the retina, we analyzed the morphology of the Sez-6 knockout mouse retina with immunohistochemical markers and compared ganglion cell dendritic arbor patterning in Sez-6 null retinae with controls. The functional importance of Sez-6 was assessed by dark-adapted paired-flash electroretinography (ERG).

**Conclusions:**

In summary, we have reported the detailed expression pattern of a novel retinal marker with broad cell specificity, useful for retinal characterization in rodent experimental models. Retinal morphology, ganglion cell dendritic branching and ERG waveforms appeared normal in the Sez-6 knockout mouse suggesting that, in spite of widespread expression of Sez-6, retinal function in the absence of Sez-6 is not affected.

## Introduction

Seizure-related gene 6 (*sez-6*) is expressed in the developing and adult mouse brain and retina [1]–[3] in addition to other neural tissues. In the developing brain, *sez-6* expression is prominent in maturing neurons of the cortical plate and cerebellum [1], [2]. Recent phenotypic characterization of Sez-6 null-mutant mouse brain has revealed a role for Sez-6 in specifying dendritic branching patterns of cortical neurons [4]. Pyramidal neurons in the cortex of *sez-6* null mice exhibit an excess of short dendrites, diminished excitatory post-synaptic responses and significantly fewer excitatory synapses [4]. Since *sez-6* is an activity-regulated mRNA transcript, up-regulated in neurons after pentylenetetrazole (PTZ) treatment [5], training mice in an enriched environment [6] and long-term potentiation induction [7], we wish to investigate whether or not Sez-6 is involved in the maturation and/or activity-regulated remodelling of retinal circuitry [8], [9].

Three isoforms of Sez-6 are present in the brain, produced from alternatively-spliced mRNA transcripts [10]. Two protein isoforms (Sez-6 type I and type II) are cell-surface proteins tethered by a single transmembrane domain while the third, Sez6 type III, is a secreted protein identical to the amino terminal sequences of type I and type II except for 18 C-terminal amino acids. All Sez-6 isoforms contain CUB (Complement sub-component C1r, C1s/sea Urchin embryonic growth factor Uegf/Bone Morphogenetic Protein 1) and SCR (Short Consensus Repeat or sushi) domains. The presence of these well-known protein-protein interaction domains suggests that Sez-6 function involves binding to other extracellular or cell-surface proteins.

The aims of this study were to characterize the cellular expression patterns of Sez-6 using a specific Sez-6 polyclonal antibody able to detect the multiple isoforms and to investigate the function of Sez-6 in the rodent retina. Using established cell-type specific markers we compared retinal morphology and composition in Sez-6 wild-type and knockout mice. Dendritic arbor patterning of retinal ganglion cells was examined in the Sez-6 knockout line crossed with a Thy1-yellow fluorescent protein (YFP) transgenic mouse line. Additionally, we compared paired-flash electroretinogram waveforms between Sez-6 wild-type and knockout mice to determine whether Sez-6 function is required for normal electrophysiological output of the retina.

## Methods

### Ethics Statement

All experimental procedures using animals were conducted according to guidelines provided by the Australian and New Zealand Council for the Care of Animals in Research and Teaching (ANZCCART) and approved by the Animal Ethics Committee of the Howard Florey Institute or the University of Melbourne.

### Animal procedures and tissue preparation

The sources of retinal tissue for immunohistochemistry were adult Sprague-Dawley rats and adult wild-type or Sez-6 knockout mouse littermates (129Sv/J×C57Bl6/J [4]). To obtain retinal tissue containing yellow fluorescent protein (YFP)-labeled ganglion cells, the Sez-6 knockout line was crossed with the Thy1-YFP line H [11]. Rats were sedated using ketamine hydrochloride (50 mg/kg body weight) and xylazine (5 mg/kg), and lethally injected with an overdose of sodium pentobarbital (60 mg/kg). Mice were killed by cervical dislocation. The eyes were rapidly enucleated, the anterior segment and vitreous removed, and the posterior eyecups placed in fixative. For immunohistochemistry, posterior eyecups were lightly fixed in 4% (w/v) paraformaldehyde in 0.1 M phosphate buffer (PB) (pH 7.4) for 15–30 minutes. Following fixation, the eyecups were rinsed in 0.1 M PB and cryoprotected in graded sucrose solutions (10%, 20% and 30%). Eyecups were vertically sectioned at 12 µm on a cryostat and collected on poly-L-lysine-coated slides.

### Production of anti-Sez6 antiserum

A cDNA encoding the mouse Sez-6 type III isoform lacking the native signal peptide (beginning at amino acid Thr27, [5]; see cDNA clone MGC:65649 IMAGE:6833344) was amplified from a SuperscriptII reverse-transcriptase (Invitrogen) generated template from mouse E15 brain using EXPAND HiFi PCR (Roche). The cDNA was cloned in-frame into the *Nhe*I/*Not*I restricted CMV-NFlag vector [12] and sequence verified. The amino terminal FLAG epitope-tagged recombinant Sez-6 tIII protein was purified from transfected Cos-7 cell conditioned medium on an anti-FLAG affinity resin and used to produce a rabbit polyclonal antiserum.

### Western blot

Western blotting was carried out as described previously [4] using protein extracts prepared in RIPA buffer with protease inhibitors (Complete Mini; Roche Diagnostics). Protein fractions (from embryonic day 15 brain or post-natal day 9 tissues; 50 µg protein/lane) were resolved using SDS-PAGE and transferred to BioTrace PVDF membranes (Pall Corporation, Port Washington, NY). Anti-Sez-6 antiserum was used at a dilution of 1/1000 as the primary antibody for detection.

### Immunohistochemistry

Anti-Sez-6 antiserum (1/5000 dilution, 3rd bleed or 1/2000 of antiserum affinity purified on a Protein G column) was used either alone or in combination with the following primary antibodies: guinea pig anti-GABA (Chemicon, Temecula, CA, USA; 1/500); mouse anti-parvalbumin (Swant, Bellinzona, Switzerland; 1/4000); mouse anti-choline acetyl transferase (ChAT) (Chemicon, Temecula, CA, USA; 1/500; used on rat retinal sections) or goat anti-ChAT (Chemicon, Temecula, CA, USA; 1/50; used on mouse sections); mouse anti-Protein Kinase C (Sigma, MO, USA; 1/400); mouse anti-Tyrosine Hydroxylase (Chemicon, Temecula, CA, USA; 1/1000); mouse anti b-NOS (Sigma, St Louis, MO, USA; 1/3000); mouse anti-calretinin for rat retinal sections or goat anti-calretinin for mouse retinal sections (CalR; both from Swant, Bellinzona, Switzerland); mouse anti-vasoactive intestinal peptide (VIP; kindly donated by Dr J Walsh and Dr H Wong, UCLA, LA, USA; 1/100), mouse anti-neurofilament 145 (MAB 1621; Chemicon, Temecula, CA, USA; 1/500) and mouse anti-GM130 as a marker of Golgi (BD Australia, North Ryde, NSW, Australia; 1/100). Secondary antibodies and tertiary reagents were biotinylated anti-rabbit (Vector Labs., Burlingame, CA; 1/200) or AlexaFluor594–conjugated donkey anti-mouse IgG (Molecular Probes, Eugene, OR; 1/500) and fluorescein-avidinD (Vector Labs.; 1/200), respectively. Nuclei were counterstained with bisbenzamide (Bis) added to the secondary antibody solution when required (2.5 µg/ml; Sigma Aldrich, NSW, Australia).

### Fluorescent Imaging and Image Analysis

Confocal images were obtained using a Zeiss LSM5 Pascal microscope equipped with argon and He/Ne lasers. Z-stacks of images were captured and co-localization of staining in double-labelled sections was examined in single optical sections of z-stack confocal images. Conventional epifluorescence microscopy was performed on bisbenzamide and Sez-6 stained sections using an Olympus BX51 upright microscope and a 40×objective. For analysis of retinal ganglion cell morphology, YFP-labelled ganglion cells were identified by the presence of an axon and neurons with the morphology of large field ganglion cells (A1, A2, C1 and C2 using the criteria of Sun et al., 2002) were selected. Dendritic arbors of ganglion cells were traced on projected z-stacks using Image Pro Plus and Sez-6 wild-type (n-22) and knockout (n = 23) populations were compared using a two-tailed Student's t-test.

### Paired-flash electroretinogram recording

The retinal function of 8 mice per genotype (6–7 months of age) was assessed by the flash electroretinogram (ERG) as described previously [13], [14]. Briefly, animals were anaesthetized following overnight dark adaptation with a mixture of ketamine and xylazine (50∶10 mg/kg). Corneas were anaesthetized with topical 0.5% proxymetacaine (Ophthetic, Allergan, Frenchs Forest, New South Wales, Australia) and pupils dilated with 0.5% tropicamide (Mydriacyl; Allergan, Frenchs Forest, New South Wales, Australia). Full-field flash ERGs were recorded with stainless steel electrodes (active – cornea, inactive – mouth, reference - tail). Responses were amplified (gain×5000; –3 dB at 1 Hz and 1 kHz, ADInstruments, USA) and digitized at 10 kHz over a 200 ms epoch. The stimulus was a commercial photographic flash unit (Metz Mecablitz 60CT4) delivered via a Ganzfeld sphere, and stimulus energy was attenuated by the addition of neutral density filters. Signals were collected over three light intensities (1.4, 1.7 and 2.0 log cd s m^–2^). Cone and rod contributions to the ERG waveform were isolated via a paired-flash protocol as described in Phipps et al. [13], [14]. This involved two flashes presented in succession with an inter-stimulus interval (ISI) of 0.8 s. The short ISI ensures that rod responses have not recovered when the second flash is presented, resulting in a cone only response to the second flash. Rod contributions were isolated by subtraction of the cone waveform from the mixed rod/cone ERG waveform (signal collected from the first flash).

### ERG component analysis

Photoreceptor function was assessed by modelling the leading edge of the a-wave (PIII model) as described previously [14], using a modified version of the Hood and Birch model [15]:
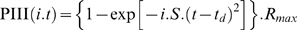
where PIII gives the summed photocurrent as a function of luminous exposure *i* (cd.s.m^−2^) and time, *t* (in seconds). R_max_ (µV) is the saturated amplitude of the PIII, *S* (sensitivity) represents the gain of the phototransduction process (m^2^.cd^−1^.s^−3^) and t_d_ (seconds) is a delay that accounts for biochemical and recording latencies following stimulation. The PIII model was fitted to the ensemble of a-waves collected (1.4–2.0 log cd.s.m^−2^) through the optimisation of R_max_ and *S* parameters. Optimisation was accomplished through minimisation of the sum-of-squares (SS) error term using the solver function of Excel™.

Characterisation of inner retinal function was achieved by examination of the PII component and the oscillatory potentials. The PII component is represented by the b-wave following extraction of the PIII, and described by its maximum amplitude (in µV) and implicit time (ms). The cone function of the mice was described by analysing the cone PII only, as the number of cones in the mouse retina is too small for reliable extraction of a cone photoreceptor (PIII) or oscillatory potential response.

Oscillatory potentials (OPs) appear on the rising slope of the rod b-wave and were isolated by removing elements that overlap with the dominant frequencies of OPs in the frequency spectrum (PIII and PII) [16]. Briefly, the PIII and the rising slope of the b-wave were digitally subtracted from the raw waveform to yield oscillations that were filtered (55–280 Hz at -3 dB, 512-tap FIR filter, Blackman window). The conditioned waveform was modelled by a Gabor in the time domain (Equation 2);
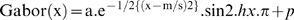
(2)


The gabor describes the OPs by the maximum amplitude (*a*, uV), time to peak (*m*, ms), spread (*s*, ms), frequency (*h*, Hz) and phase (*p*). Data extraction and fitting was achieved by floating all parameters and minimizing the sum-of-squares error term in an Excel^TM^ spreadsheet.

### Statistical Analysis

Data were analyzed (SigmaStat for Windows, ver. 3.10; Systat Software Inc, Point Richmond, CA), and a one-way ANOVA with a Tukey post hoc comparison was applied, with *P*<0.05 considered statistically significant for homogenous and normally distributed data. In cases of non-normal or nonhomogenous data, a Kruskal-Wallis test was applied with the Dunn post hoc comparison, and *P*<0.05 was considered statistically significant.

## Results

**Figure 1:**
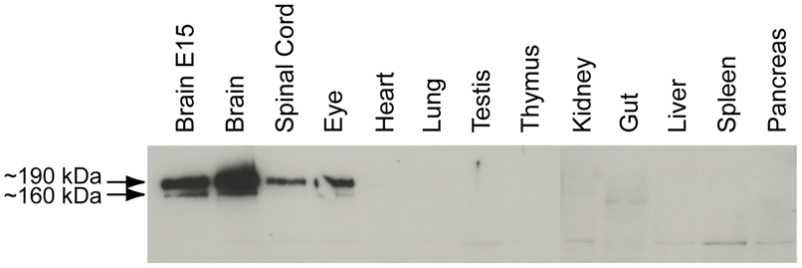
Sez-6 is expressed in the brain, spinal cord and eye. Western blot of tissues (50 µg protein/lane) from postnatal day 9 (P9) mice and embryonic brain extract (embryonic day 15), using anti-Sez-6 antiserum (1/1000) as the primary antibody for detection.

The tissue and cellular specificity of Sez-6 protein expression has not been examined previously due to the lack of a specific antibody. We raised a polyclonal antibody in rabbit to the secreted isoform of Sez-6 (tIII; identical to the amino terminus of tI and tII isoforms except for 18 amino acids at the C-terminal end) in order to address these questions. Sez-6 is exclusively expressed in neural tissues, including embryonic day 15 (E15) and adult brain, spinal cord and eye, as determined by Western blot (Fig. 1). Band sizes of 160 kDa and 190 kDa were consistently detected and are likely to represent glycosylated Sez-6 isoforms with the larger band corresponding to Type I/II [10].

**Figure 2:**
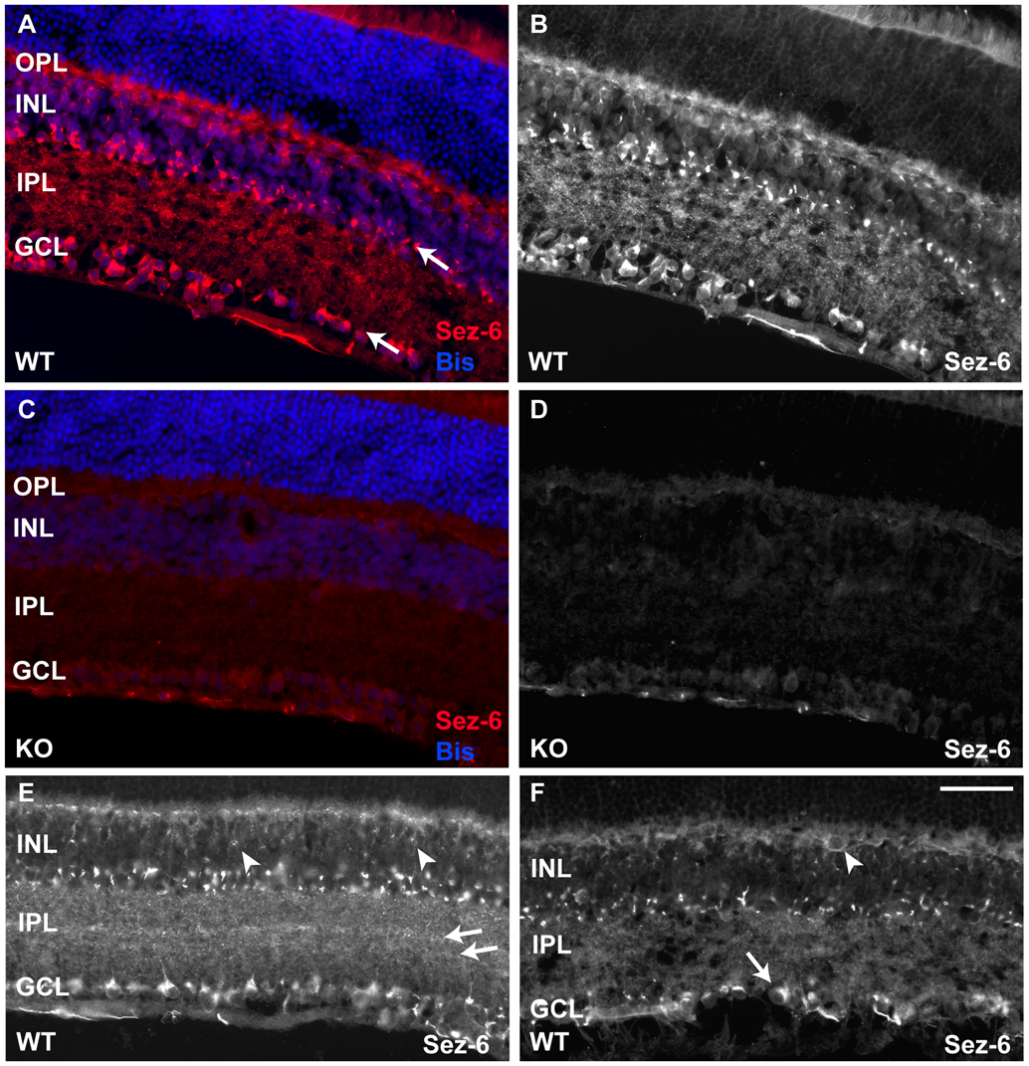
Immunostaining for Sez-6 in the mouse retina reveals labelling in all layers except the outer nuclear layer. (A) Sez-6 immunostained wild-type mouse retinal section counterstained with bisbenzamide to reveal the morphology of nuclear layers. Layers displaying the strongest staining for Sez-6 are marked by arrows. (B) Sez-6 immunostaining alone on the wild-type section shown in A. (C) Sez-6 knockout (KO) mouse retina immunostained with anti-Sez-6 antiserum and counter-stained with bisbenzamide indicating a low level of non-specific staining in the GCL, INL, OPL and photoreceptor outer segments. The Sez-6 staining alone on the same KO section is shown in (D). (E) Wild-type retina stained for Sez-6 with examples of more weakly stained bipolar cell somata in the INL indicated (arrowheads). (F) Wild-type retina stained for Sez-6 with cells morphologically resembling a horizontal cell and an alpha (or A1) ganglion cell indicated (arrowhead and arrow, respectively). Scale Bar = 50 µm.

Initially, we tested the rabbit anti-Sez-6 antiserum on sections of adult mouse retina (Fig. 2) utilizing equivalent retinal sections from Sez-6 null mice to determine the level of non-specific staining. In wild-type retina, specific Sez-6 immunostaining was observed in the outer plexiform layer (OPL), the inner nuclear layer (INL), the inner plexiform layer (IPL) and the ganglion cell layer (GCL) (Fig. 2A, B). Sez-6 immunoreactivity was particularly prominent in the amacrine cells of the INL (Fig. 2A, upper arrow) and stained cells in the GCL (many of which are likely to be displaced amacrines; Fig. 2A, lower arrow). In these cells, Sez-6 immunoreactivity was observed in bright puncta, frequently positioned adjacent to the nucleus and the apical dendrite. Much finer punctate staining was often observed extending into the IPL along the apical dendrites of these neurons. Nerve fiber staining on the inner side of the ganglion cell layer was considered to be non-specific as similar staining was observed in the Sez-6 knockout retina (Fig. 2C, D). Similarly, a low level of diffuse non-specific staining was observed in ganglion cells, the IPL, the OPL and photoreceptor outer segments (Fig. 2C, D).

In the IPL, specific Sez-6 staining was most prominent in two central strata (Fig. 2E, arrows) although the fine punctate staining was observed throughout. In the INL, the strongest Sez-6 immunoreactivity was observed in two rows of cells adjacent to the IPL (Fig. 2A, B, E & F) and, as mentioned above, the signal was concentrated strongly within a discrete perinuclear “bright spot” although more diffuse cytoplasmic staining was also observed in amacrine cells. Similar cellular staining was seen in retinal neurons in the outer part of the INL (Figs. 2E & F) where bipolar cell and horizontal cell somata are known to be located. Both these cell types (identified based on morphological criteria and position in the INL) appeared positive for Sez-6 (arrowheads, Figs. 2E & F) although the intensity of both the diffuse staining and the “bright spot” was lower than the levels in amacrine cells. In addition, a sub-population of retinal ganglion cells morphologically similar to alpha RGC's (or subtype A1 in the classification of Sun et al. [17]) was detected with the Sez-6 antibody (arrow in Fig. 2F;). Interestingly, the discrete brighter staining in bipolar and ganglion cells was also positioned in an apical orientation with respect to the nucleus. Bipolar cell apical dendrites were positive for Sez-6 and the OPL was stained significantly more in wild-type retina than the low level non-specific staining observed in this layer in Sez-6 knockout retina (Fig. 2A,E cf. 2C). The distinct perinuclear staining pattern of Sez-6 in amacrine and bipolar cells was reminiscent of that observed by others using an antibody to the 58 kDa resident protein of the trans-Golgi network [18]. To determine whether Sez-6 was localized to the Golgi apparatus, we conducted double-staining for Sez-6 and a marker of the cis-Golgi network, GM130 [19]. Although labelling was observed in essentially the same cell populations, we observed little sub-cellular colocalization of these two markers (data not shown).

**Figure 3:**
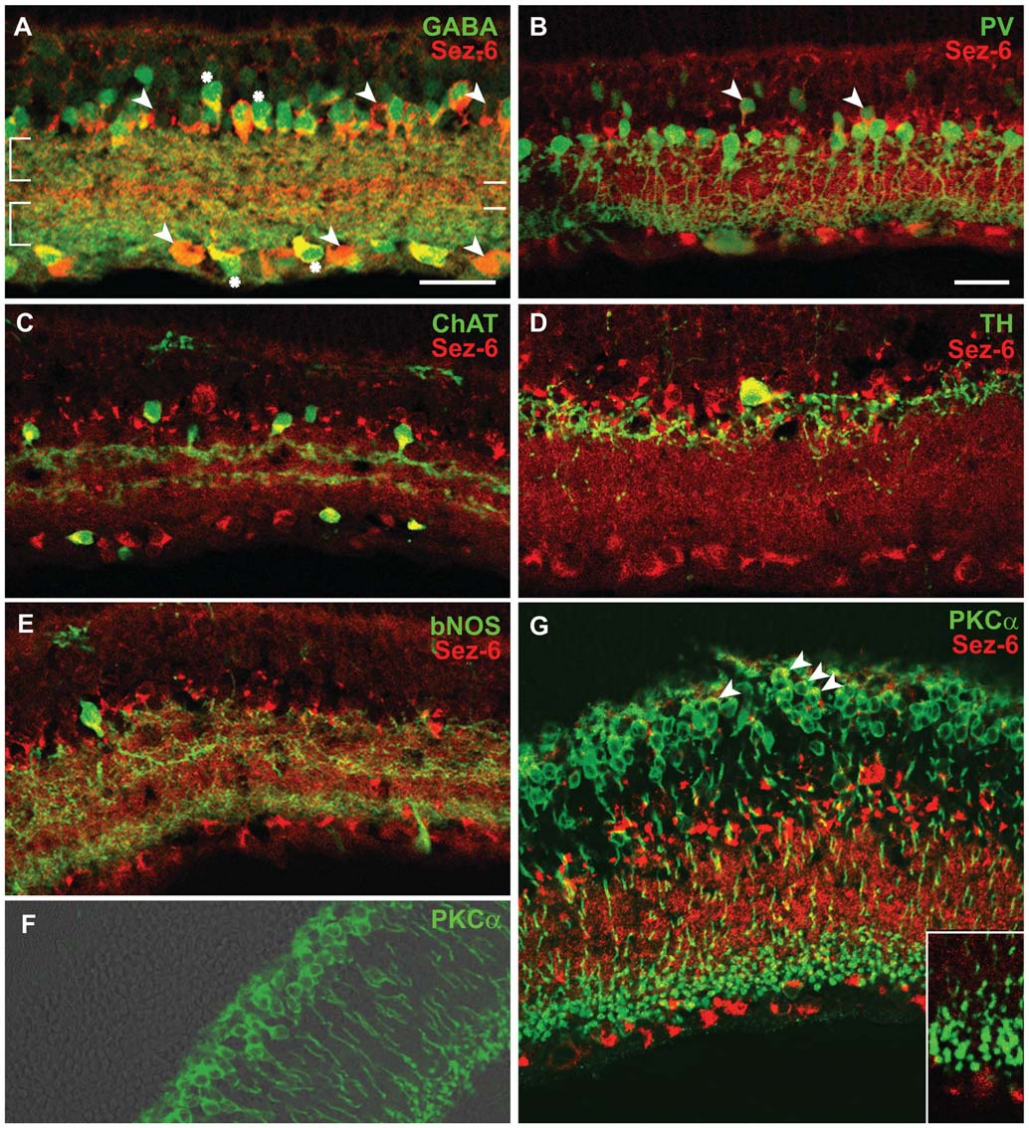
Immunostaining for Sez-6 and retinal subtype markers. Rat retina vertical sections double stained for Sez-6 (red) and (in green): (A) GABA – non-GABAergic amacrine and putative displaced amacrine cells are marked (arrowheads) while double-labelled amacrine cells are marked with asterisks. (B) Parvalbumin (PV) – Sez-6 positive amacrine cells with their somata located in the second and third layer from the IPL (arrowheads) and double-stained cells with the elongated soma typical of bipolar cells (arrows) are observed. (C) Choline acetyltransferase (ChAT)-positive amacrines in the INL and displaced amacrines in the GCL were double-labelled with Sez-6. (D) Dopaminergic amacrine cells (labelled with tyrosine hydroxylase) were rarely found however they co-labelled for Sez-6. (E) Brain nitric oxide synthase (bNOS) amacrine cells were also positive for Sez6 staining. (F & G) Immunoreactivity for Protein kinase C alpha (PKCα - green) in rod bipolar cells overlaid on a bright field image (F) or shown with Sez-6 in red (G) indicating Sez6 staining at the apical pole of the cell soma (arrowheads). Scale bars: 25 µm.

Further characterization of Sez-6 expression was subsequently carried out in the rat retina to facilitate double-immunostaining of Sez-6 with a range of well-characterized markers that are unique to specific retinal subpopulations. In rat retinal sections immunostained for GABA and Sez-6, a significant proportion of amacrines in the INL and displaced amacrines in the GCL exhibited double staining (Fig. 3A, asterisks). Interestingly, while all GABAergic amacrines in the INL were Sez-6 positive, we observed Sez-6 immunoreactive amacrines in the INL that were GABA negative and, in some cases these appeared to be aligned with Sez-6 positive/GABA negative cells in the GCL (Fig. 3A, arrowheads). Some cells in the middle to upper region of the INL displayed weaker GABA staining and these more weakly-staining neurons were also Sez-6 positive. In the IPL, two broad bands of GABA-immunoreactive processes were observed (Fig. 3A, square brackets). The inner band was found to overlay one of the distinct Sez-6 positive strata (Fig. 3A, lower horizontal bar) while the second stratum displaying more intense Sez-6 reactivity was flanked by the GABA-positive regions (Fig. 3A, upper horizontal bar). Thus, there are neuronal processes containing Sez-6 that are clearly GABA-negative, consistent with our observation that some amacrine cell somata show the same profile.

In the rat, parvalbumin (PV) is a marker for the AII subtype of glycinergic amacrine cells [20]. In rat sections double-stained for parvalbumin and Sez-6, we observed no colocalization of these markers in amacrine cells with large somata positioned immediately adjacent to the IPL (Fig. 3B). These parvalbumin-positive neurons displayed small-field dendritic trees and showed varicosities in the outer IPL characteristic of AII amacrine cells. We did observe some smaller-bodied cells with somata located in the second or third layer from the IPL that were positive for both parvalbumin and Sez-6 and extended apical dendrites towards the IPL (Fig. 3B, arrowheads).

Since the majority of Sez-6 expressing amacrines were GABAergic, we focussed on this population and utilized other markers to identify sub-populations of this class. Choline acetyltransferase (ChAT), which labels cholinergic amacrine cells in the INL and displaced amacrine cells in the GCL, showed strong co-localization with Sez-6 (Fig. 3C). Similarly, sparsely distributed dopaminergic amacrine cells labelled with tyrosine hydroxylase (TH; Fig. 3D) and brain nitric oxide synthase (bNOS; Fig. 3E) were also strongly immunoreactive for Sez-6.

Sez-6 expression is most prominent in amacrine cells although lower levels of staining are observed in other cells in the INL. We investigated whether Sez-6 is expressed in rod bipolar cells using an antibody to Protein kinase C alpha (PKCα). This antibody labelled cell somata in the outer part of the INL (Fig. 3F), the expected location for rod bipolar cells, as well as descending axons and axon terminals in the innermost part of the IPL [21]. Co-localization with Sez-6 was observed, mainly at the apical pole of the cell soma (Fig. 3G, arrowheads), although it was not seen in the axons and terminals (single optical section shown in Fig. 3G inset).

### Retinal phenotypic analysis of Sez-6 null mice

Having established the cellular expression pattern of Sez-6 in the rat retina, we carried out morphological, immunohistochemical and electrophysiological analyses of Sez-6 knockout mouse retina. This line is maintained as an intercross (129sv/J×C57Bl/6) so experimental Sez-6 null animals were obtained from heterozygous matings and wild-type littermates served as control animals.

**Figure 4:**
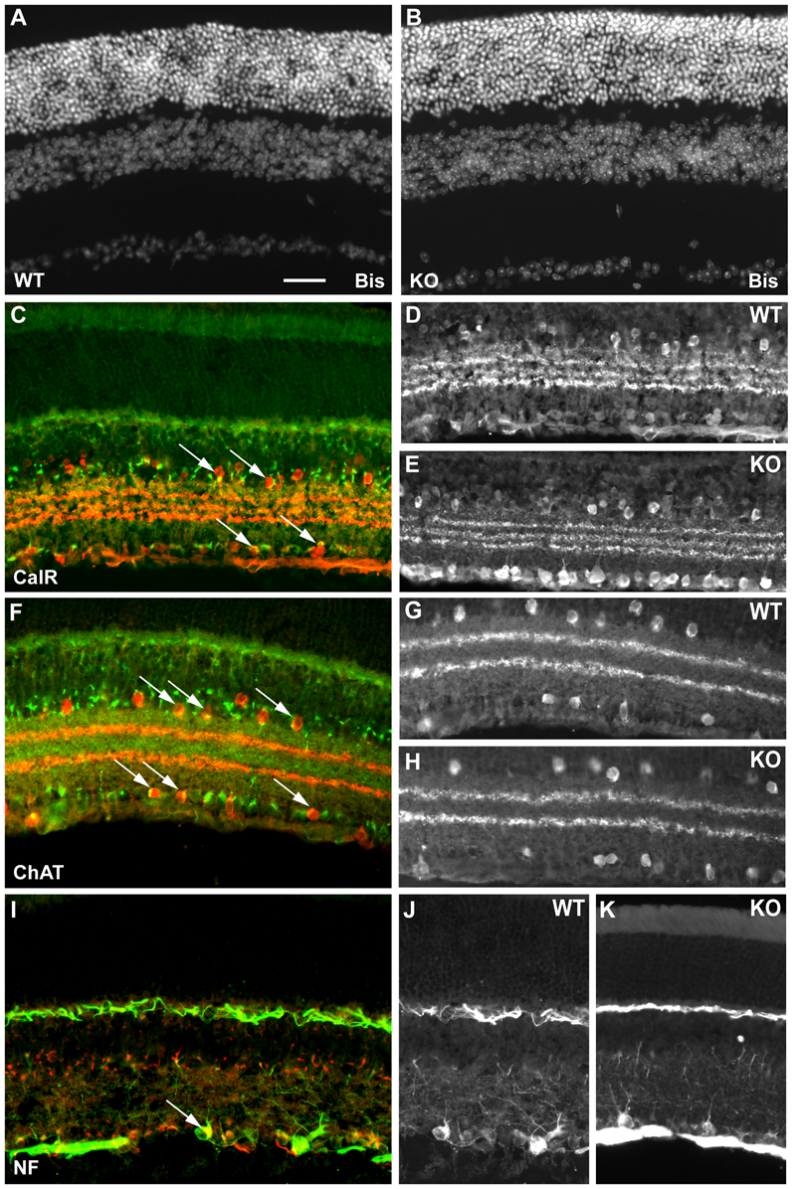
Immunostaining of Sez-6 wild-type and knockout mouse retina. Vertical sections of Sez-6 WT (A) and KO (B) mouse retina stained with bisbenzamide (Bis) reveal no obvious morphological differences between the genotypes. (C) Sez-6 WT retina stained for calretinin (CalR – red) and Sez-6 (green) showing double stained amacrine and displaced amacrine cells in the INL and GCL (arrows). (D) Sez-6 WT and (E) KO sections from the central retina stained for CalR displaying a typical staining pattern. (F) Sez-6 WT retina stained for ChAT (red) and Sez-6 (green). Double-labelled cells in the INL and GCL are indicated (arrows). (G) Sez-6 WT and (H) KO retina stained for ChAT alone show an identical staining profile. (I) Sez-6 WT retina stained for neurofilament (NF - green) and Sez-6 (red). A double-stained cell morphologically similar to an alpha (or A1) ganglion cell is marked (arrow). (J) Sez-6 WT and (K) KO retinal sections stained for neurofilament alone showing a similar density of labelled cells in the GCL in both genotypes. Scale bar = 25 µm.

Bisbenzamide staining of nuclear DNA was performed to ascertain whether there were any gross morphological abnormalities in retinal organization. Sections from Sez-6 wild-type (WT) and knockout (KO) central retinae were compared from 6 animals of each genotype. The cellular organization of Sez-6 null retina and the thickness of the nuclear layers were not apparently different from those of wild-type retina (Fig. 4A & B).

In wild-type mice, calretinin labels a sub-population of GABAergic amacrine cells in the INL along with displaced amacrines in the GCL [22]. In double-labelled sections for Sez-6 and calretinin, some Sez-6 labelling of calretinin-expressing amacrine cells was observed (Fig. 4C, arrows). Comparison of wild-type and Sez-6 knockout retinae immunostained for calretinin alone revealed no obvious differences: the characteristic staining of calretinin-positive processes in three distinct strata in the IPL was observed in both wild-type and Sez-6 knockout sections (compared in vertical sections of the central retina; Fig. 4D & E; note that the increased staining in the RGC layer in Fig. 4 E was not typical of other sections – not shown).

In wild-type sections immunostained for ChAT and Sez-6, we observed a high degree of co-labelling as described previously (arrows in Fig. 4F) although comparison of ChAT-stained sections from wild-type and Sez-6 knockout revealed no detectable differences in ChAT^+^ neuron number, distribution or stratification of processes (Fig. 4G & H). In sections stained for neurofilament (NF), strong staining was seen in ganglion cell axons in the nerve fibre layer and neuronal processes in the OPL as well as in large neuronal somata and their apical dendrites projecting into the IPL. These cells were similar in location and morphology to alpha ganglion (or A1) cells and they occasionally double-stained in wild-type sections with the Sez-6 antibody (Fig. 4I, arrow). These cells were observed in similar numbers in sections of the Sez-6 knockout retina and the NF staining appeared highly similar in sections of both genotypes (Fig. 4 J & K).

**Figure 5:**
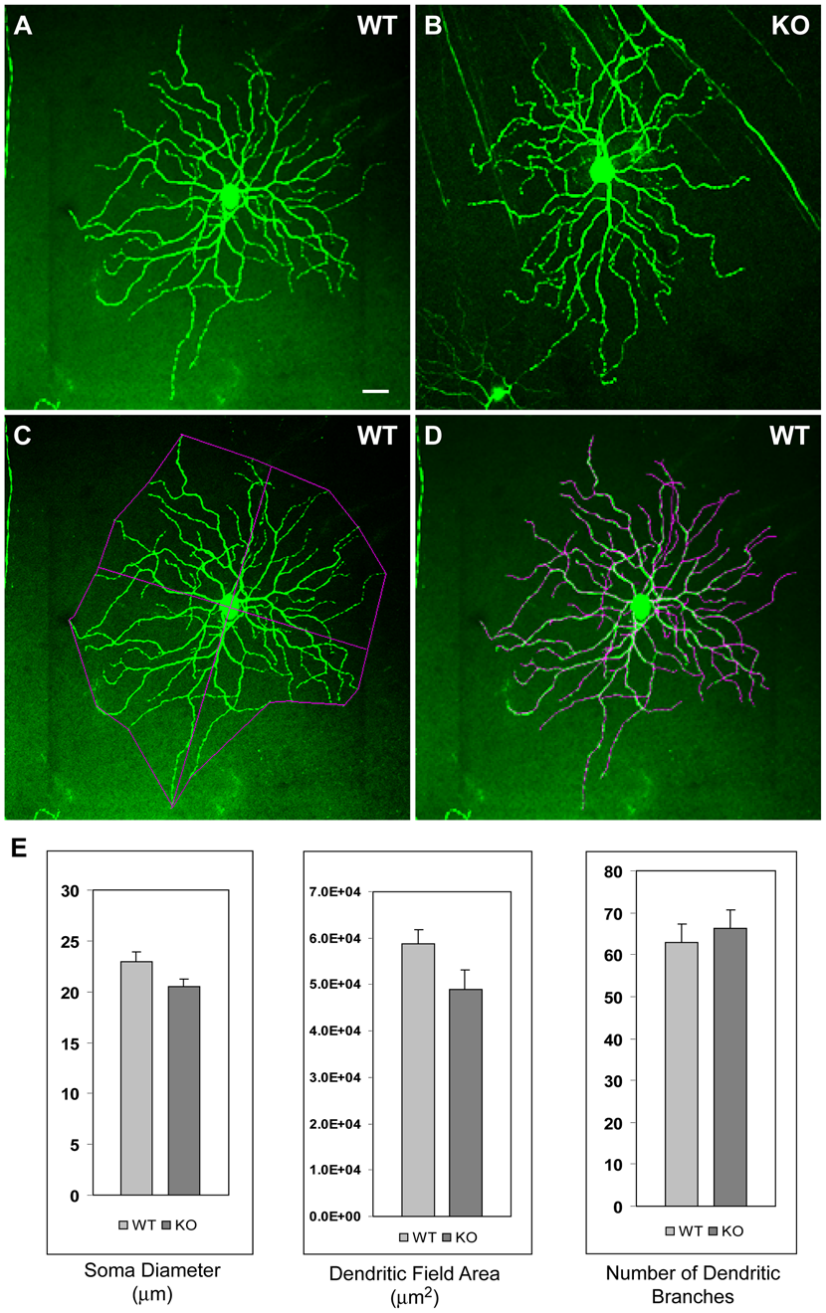
Dendritic arborization of retinal ganglion cells in wild-type and Sez-6 null retina. Projected z-stacks of YFP-labelled large field ganglion cells in WT (A) and KO (B) mouse retina. Dendritic field area was measured as shown in (C) and an example of dendrite tracing is shown in (D). Soma diameter, dendritic field area and branch number did not differ between WT (n = 22) and KO (n = 23) neurons (E).

Although we detected no obvious morphological differences between wild-type and Sez-6 null retinae, it was possible that relatively subtle alterations (for example, in dendritic arborization) may not have been detected in vertical retinal sections. To address this possibility, we took advantage of a mouse line in which retinal cells (predominantly retinal ganglion cells) are labelled by transgenic expression of YFP (Thy1-YFP line H [11]). Since Sez-6 is expressed in bipolar cells and retinal ganglion cells as well as amacrine cells, we reasoned that lack of Sez-6 could affect the extent and/or branching of ganglion cell dendritic arbors by altering inputs to these cells. We analysed dendritic arborization of retinal ganglion cells (labelled with YFP) in flat mounted retinae from Sez-6 WT and KO mice crossed with Thy1-YFP line H (Fig. 5A & B; 6 retinae per genotype). Retinal ganglion cells were identified by the presence of an axon and neurons with the morphology of large field ganglion cells (A1, A2, C1 and C2 using the criteria of Sun et al. [17]) were scored. The area of the dendritic field was determined as the area of a polygon obtained by joining the distal dendritic extremities (Fig. 5C) and dendrites were traced to determine branch number (Fig. 5D). As shown in Fig. 5E, there was no difference in morphological parameters (soma diameter, field area or branch number) between RGC's in Sez-6 WT and KO mouse retinae.

To investigate whether Sez-6 gene ablation affected retinal neuron function, we conducted paired-flash dark-adapted electroretinograms (ERGs) on a cohort of 8 age-matched mice per genotype. The ERG components were modelled as described in Materials and Methods (Table 1). No significant differences between the Sez-6 knockout group and the wild-type controls were observed in any of the ERG waveform components suggesting that, in these knockout mice, the lack of Sez-6 has no appreciable effect on retinal function.

**Table 1:** Paired-flash dark-adapted electroretinogram components in wild-type and Sez-6 knockout mice.

ERG parameter		Sez-6 WT (±SEM)	Sez-6 KO (±SEM)	p-value
***Rod a-wave***	**R_max_(µV)**	257.4±34.7	284.7±29.4	0.58
	**log S (m^2^.cd^−1^.s^−3^)**	3.5±0.1	3.4±0.1	0.60
**Rod b-wave**	**PII amplitude (µV)**	405.7±81.3	539.9±31.2	0.22
	**Peak time (ms)**	78.1±9.8	93.0±4.5	0.79
**Cone b-wave**	**Cone PII amplitude (µV)**	226.6±23.9	259.7±21.6	0.33
	**Cone PII peak time (ms)**	49.7±2.9	48.7±3.0	0.82
**Oscillatory Potentials**	**Amplitude (µV)**	59.0±13.2	77.7±7.5	0.22
	**Time to peak (ms)**	30.9±2.1	30.2±1.4	0.78
	**Spread (ms)**	6.9±1.5	6.4±1.0	0.75
	**Frequency (Hz)**	108.4±1.4	100.2±5.3	0.23

## Discussion

We report here the expression pattern of Sez-6 in the rodent retina. Sez-6 is expressed most strongly in amacrine cells and labels most, if not all, GABAergic amacrines with a characteristic punctate pattern. This distribution of Sez-6 immunostaining, in an intense spot positioned apically to the nucleus, was reminiscent of the pattern obtained with the cis-Golgi apparatus marker, GM130 [19] although we did not observe overlap of the Sez-6 staining pattern with that of GM-130. A very similar apical “cap” staining pattern has also been reported for soluble guanylate cyclase in cortical pyramidal neurons [23]. The significance of this observation is not clear although it is interesting to note that soluble guanylate cyclase/cyclic GMP signaling is involved in neuron polarization and oriented dendritic outgrowth during development.

As described above, not all Sez-6 positive amacrine cells were GABAergic. Additionally, the central region of the IPL contained Sez-6 positive processes flanked by the two broad strata labelled with GABA. From these observations, we conclude that Sez-6 also labels a population of glycinergic amacrine cells. As AII amacrine cells are the most abundant glycinergic subtype in the rat retina [24], we investigated whether Sez-6 was expressed in this subtype (marked by parvalbumin staining) however we observed no co-localization of Sez-6 and parvalbumin in AII amacrine cells. Nevertheless, Sez-6 may serve as a useful marker of amacrine cells in the rodent retina as it is expressed by the majority of GABAergic amacrine subtypes and some (non-AII) glycinergic amacrine cells. Additionally, the Sez-6 antibody weakly labelled other neuronal cell types including bipolar and horizontal cells although specific Sez-6 immunostaining was notably absent from photoreceptors.

Given the range of retinal cell types expressing Sez-6 and the role Sez-6 plays in shaping the dendritic arbor and excitatory connectivity in cortical pyramidal neurons [4], it was surprising that ERG analyses failed to reveal any functional consequences of Sez-6 absence in the retina. As a mass electrical potential, the ERG trace represents the summed activity of the entire retinal circuitry. It is therefore possible that single cell electrophysiological analysis would be required to detect putative functional abnormalities in subsets of amacrine cells that may arise from Sez-6 gene inactivation. Examples in the literature of normal ERG responses in gene knockout mice include the G-substrate [25], mouse retinal degeneration B2 (rdgB2; [26]) and transthyretin [27] null mouse lines. Like Sez-6, G-substrate and rdgB2 are expressed in subsets of amacrine and ganglion cells. The normal ERG responses in the rdgB2 and transthyretin knockout mice were attributed to likely functional compensation mechanisms and this explanation could well apply to the results obtained here. Sez-6 is a member of a gene family and the two closest family members (Sez6L and Sez-6 L2) are also expressed in eye (according to expressed sequence tag expression profiles and the GENSAT database). The developmental profiles of Sez-6 and Sez6L2 mRNA expression in the retina, revealed by in situ hybridization, are highly similar (see Table S5 Ref. [3]; Sez6L2 is also known as Psk-1). Therefore, it seems likely that one or more Sez-6 family members can functionally compensate for the lack of Sez-6 in the retina.

Activity-mediated interactions between RGCs and bipolar and amacrine cells are known to be important for maturation of the RGC dendritic arbor, including the pruning of branches to form ON or OFF sublaminae in the IPL [28], [29], [8]. Given the expression of Sez-6 in amacrine cells as well as weaker expression in bipolar and ganglion cells, we had hypothesized that the dendritic patterning of the ganglion cells themselves may be altered in Sez-6 knockout mouse retina. We were unable to detect any gross abnormalities in retinal morphology or in the dendritic arborization patterns of large-field retinal ganglion cells in Sez-6 knockout mice. In this study, we did not specifically examine whether the developmental pruning of bistratified to monostratified RGC dendritic arbors might be delayed [8] although this question would be well worth investigating in future [9].

In conclusion, we have determined that Sez-6 is broadly expressed in the retina with a strong and characteristic staining pattern in amacrine cells. Despite this widespread expression, the absence of Sez-6 did not appear to result in morphological or functional abnormalities. Future studies that address Sez-6 redundancy and functional compensation by other members of the gene family will throw additional light on the function of Sez-6.
